# Pre‐Emptive Amlodipine for FOLFIRINOX‐Induced Hypertensive Crises Without Primary Hypertension: A Novel Chronotherapeutic Strategy

**DOI:** 10.1002/ccr3.72527

**Published:** 2026-04-14

**Authors:** Manas Pustake, William Wells‐Gatnik, Vani Shukla, Javier Corral, Sumit Gaur

**Affiliations:** ^1^ Department of Internal Medicine Texas Tech University Health Sciences Center El Paso Texas USA; ^2^ Department of Hematology and Oncology Texas Tech University Health Sciences Center El Paso Texas USA

**Keywords:** adverse event management, amlodipine, calcium channel blocker, chemotherapy‐induced hypertension, chronotherapy, FOLFIRINOX, oncology supportive care, pancreatic cancer

## Abstract

FOLFIRINOX remains a cornerstone therapy for advanced pancreatic ductal adenocarcinoma (PDAC), but its toxicity profile extends beyond myelosuppression and neuropathy to include even rarer complications such as treatment‐associated hypertension. Infusion‐related hypertensive episodes represent a clinically challenging and underrecognized complication, as conventional daily antihypertensive strategies may control acute elevations yet precipitate inter‐cycle hypotension. We describe a 46‐year‐old woman with metastatic PDAC who developed recurrent hypertensive crises (peak 190/102 mmHg) temporally associated with FOLFIRINOX infusions. A trial of daily losartan (50 mg) mitigated infusion‐related hypertension but resulted in symptomatic inter‐cycle hypotension, limiting dose escalation. To better align antihypertensive therapy with the predictable timing of blood pressure surges, a chronotherapeutic strategy was implemented using low‐dose daily losartan (25 mg) combined with a single pre‐emptive dose of amlodipine (5 mg) administered 8–12 h prior to chemotherapy infusion after informed decision‐making. This approach resulted in consistent control of infusion‐related blood pressure elevations (systolic ~125–145 mmHg during treatment), elimination of hypertensive crises, and maintenance of stable inter‐cycle hemodynamics without symptomatic hypotension. Importantly, this strategy enabled uninterrupted delivery of full‐dose chemotherapy. This case highlights a novel, hypothesis‐generating chronotherapeutic approach to chemotherapy‐induced hypertension. Timed antihypertensive administration may represent a targeted strategy to control infusion‐related blood pressure surges while preserving baseline hemodynamic stability; however, further validation in larger studies is required before clinical generalization.

## Key Clinical Message

FOLFIRINOX and other chemotherapy regimens may cause infusion related hypertensive crises less focused on in current guidelines. In patients who develop inter cycle hypotension with daily antihypertensives, timed pre infusion amlodipine can control chemotherapy associated blood pressure surges while preserving baseline hemodynamic stability and enabling uninterrupted treatment delivery.

## 1 Introduction

Pancreatic ductal adenocarcinoma (PDAC) carries one of the poorest prognoses in oncology, with FOLFIRINOX representing the standard first‐line palliative therapy for patients with adequate performance status. While this regimen demonstrates superior efficacy compared to gemcitabine monotherapy, its toxicity profile remains formidable; Grade 3–4 adverse events occur in approximately 75%–80% of patients, with neutropenia affecting 45.7%, fatigue 23.6%, and severe diarrhea 12.7% of treated individuals [1] Beyond these well‐characterized toxicities, cardiovascular complications, particularly arterial hypertension and hypertensive crises, may occur and remain insufficiently characterized in the literature.

The phenomenon of cancer therapy‐induced hypertension (CTIH) during FOLFIRINOX administration presents unique management challenges that current oncology guidelines do not address in detail. In current guideline‐based supportive care for patients receiving FOLFIRINOX, emphasis is placed on expected toxicities such as nausea/vomiting, diarrhea, neutropenia/febrile neutropenia, and neuropathy; hypertensive crisis is not described as a typical or frequent FOLFIRINOX toxicity in the major pancreatic‐cancer and supportive‐care sources [1, 2]. This pattern often involves acute hypertensive episodes during infusion periods, followed by normotension or relative hypotension during intercycle intervals. Standard daily antihypertensive regimens may be poorly suited to this pattern, often causing deleterious hypotension between treatments while failing to control infusion‐related surges.

The pathophysiology underlying FOLFIRINOX‐induced hypertension likely involves multiple converging mechanisms. 5 fluorouracil may induce endothelial dysfunction through nitric oxide depletion and direct vasospastic effects [3]. Irinotecan may disrupt autonomic regulation, while oxaliplatin may contribute to vascular toxicity through platinum‐related endothelial injury [4, 5]. Dexamethasone, a potent synthetic glucocorticoid, is commonly administered prior to chemotherapy to prevent nausea, vomiting, and hypersensitivity reactions. Glucocorticoids can contribute to transient blood pressure elevation through several mechanisms, including enhanced vascular sensitivity to catecholamines, mild sodium and water retention, and modulation of endothelial nitric oxide signaling [6]. In the context of FOLFIRINOX administration, dexamethasone may therefore act as an amplifying factor that augments the hypertensive effects of chemotherapy‐induced endothelial dysfunction and autonomic imbalance. However, corticosteroid premedication alone rarely produces abrupt hypertensive crises in otherwise normotensive patients; rather, its effects are typically modest and temporally limited. Accordingly, we propose that dexamethasone may function as a permissive or synergistic contributor within a broader multifactorial mechanism involving fluoropyrimidine‐associated endothelial injury, irinotecan‐related autonomic dysregulation, and oxaliplatin‐mediated vascular toxicity. This combined physiologic stress may lower the threshold for acute infusion‐related hypertensive surges during chemotherapy administration.

The management of acute infusion related hypertensive surges presents a distinct therapeutic challenge, as conventional daily antihypertensives risk inducing inter‐cycle hypotension. This limitation raises the possibility of applying chronotherapeutic strategies, well established in cardiovascular medicine for aligning drug administration with circadian variation in blood pressure regulation, to oncology supportive care [7, 8]. Circadian variation in blood pressure regulation, mediated through fluctuations in sympathetic tone and renin angiotensin aldosterone system activity, may be further disrupted by chemotherapy related endothelial injury and inflammatory signaling. These observations support consideration of time directed antihypertensive therapy in selected patients.

We present a case of severe, recurrent hypertensive crises temporally linked to FOLFIRINOX infusions that proved refractory to conventional management strategies. Through implementation of a preemptive amlodipine dosing strategy administered 8 to 12 h before chemotherapy infusion, we achieved consistent blood pressure control while maintaining inter cycle hemodynamic stability. This approach enabled uninterrupted delivery of full dose chemotherapy without delays or dose reductions. To our knowledge, no prior reports describe prophylactic timed calcium channel blockade for the prevention of FOLFIRINOX associated hypertensive crises.

## 2 Case History/Examination

A 46‐year‐old woman with a history of well‐controlled asthma presented with right upper quadrant pain, pruritus, and abnormal liver function tests. Subsequent imaging revealed a hypoenhancing mass in the head of the pancreas along with a malignant stricture of the common bile duct. A diagnosis of invasive ductal adenocarcinoma was confirmed via endoscopic ultrasound‐guided biopsy. Staging work‐up identified two metastatic liver lesions. Her baseline CA 19–9 was significantly elevated at 16,770 U/mL. Following an ERCP with placement of a covered metal stent, she was diagnosed with stage IV pancreatic ductal adenocarcinoma and started on palliative FOLFIRINOX. She had no prior personal history of hypertension. Her baseline blood pressure prior to initiation of chemotherapy ranged between 120–130/70–80 mmHg with no prior diagnosis of hypertension or use of antihypertensive medications.

She tolerated the first two cycles remarkably well, reporting only mild, transient nausea, fatigue, and occasional loose stools. She maintained her full activity level, appetite, and ability to perform all daily activities. After eight cycles, serial imaging showed a reduction in the size of both the primary pancreatic mass and the liver metastases, coupled with a significant drop in her CA 19–9 level, confirming a positive response to treatment. Her medications included short‐course dexamethasone for antiemesis around infusion days and pancreatic enzyme replacements (Creon).

After cycle 2, we began to notice a pattern of episodic blood pressure elevations, typically into the 140–150 s/90s mmHg, that occurred specifically on the days of her chemotherapy infusions. On one occasion, the patient needed to visit the emergency department with blood pressure of 190/102 mmHg and heart rate of 118 bpm. She was monitored in the emergency department for several hours, during which her blood pressure initially improved to 136/87 mmHg with heart rate 98 bpm, but later rose again to 166/99 mmHg with heart rate 113 bpm after 6 h. She received a 1 liter intravenous normal saline bolus for tachycardia and acetaminophen for nonspecific pain. Blood pressure and heart rate gradually stabilized without the need for intravenous antihypertensive medication. At this point, she was started on Losartan 50 mg daily for hypertension.

A subsequent hypertensive episode occurred on the day of cycle 10, when the patient developed dizziness, nausea, and palpitations with blood pressure measuring 185/97 mmHg and heart rate 131 bpm; the episode resolved spontaneously, and a 1 liter normal saline bolus administered in the emergency department normalized her heart rate within approximately 4 h. Intervening chemotherapy cycles were otherwise clinically uneventful. During this time, the patient was on daily losartan.

During these episodes, she sometimes reported a sensation of “heaviness” in her head accompanied by dizziness, nausea, and palpitations, but consistently denied any chest pain, visual changes, or focal neurologic symptoms suggestive of end‐organ damage. Her blood pressure consistently returned to normal between chemotherapy cycles.

## 3 Differential Diagnosis and Investigations

We performed a systematic evaluation to exclude secondary causes of hypertension (Table 1).

**TABLE 1:** Differentials of the case and how they were ruled out.

Volume Status/Renal: Her physical exam showed no signs of edema or fluid overload; serial creatinine and eGFR remained stable within normal limits; urinalysis showed no protein or blood. Electrolytes: Sodium, potassium, calcium, and magnesium levels were consistently normal. Endocrine: She had no clinical features suggestive of hyperthyroidism, Cushing's syndrome, or pheochromocytoma (specifically, no palpitations, diaphoresis, or severe headaches). Medications: Aside from the short‐course dexamethasone taken only on infusion days, she was not using any NSAIDs, decongestants, or other known pressor agents. Other Factors: There were no significant pain or anxiety episodes coinciding with the BP spikes, no history of sleep apnea, and no evidence of acute infection or withdrawal.

**FIGURE 1:**
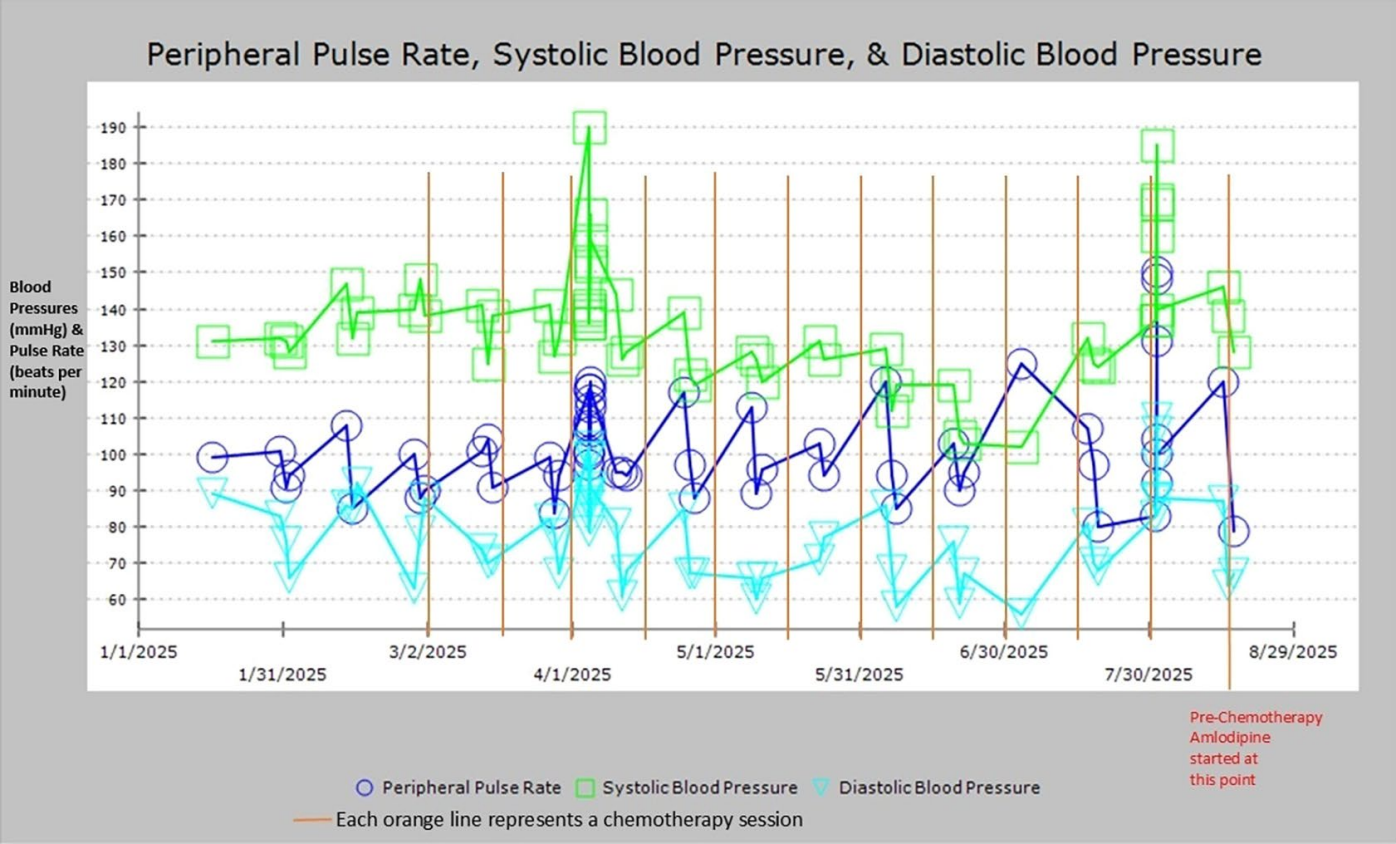
Infusion center blood pressure records during the infusions. X axis represents chronological infusion dates. Y axis represents blood pressure (systolic and diastolic) in mmHg and heart rate in beats per minute.

**FIGURE 2:**
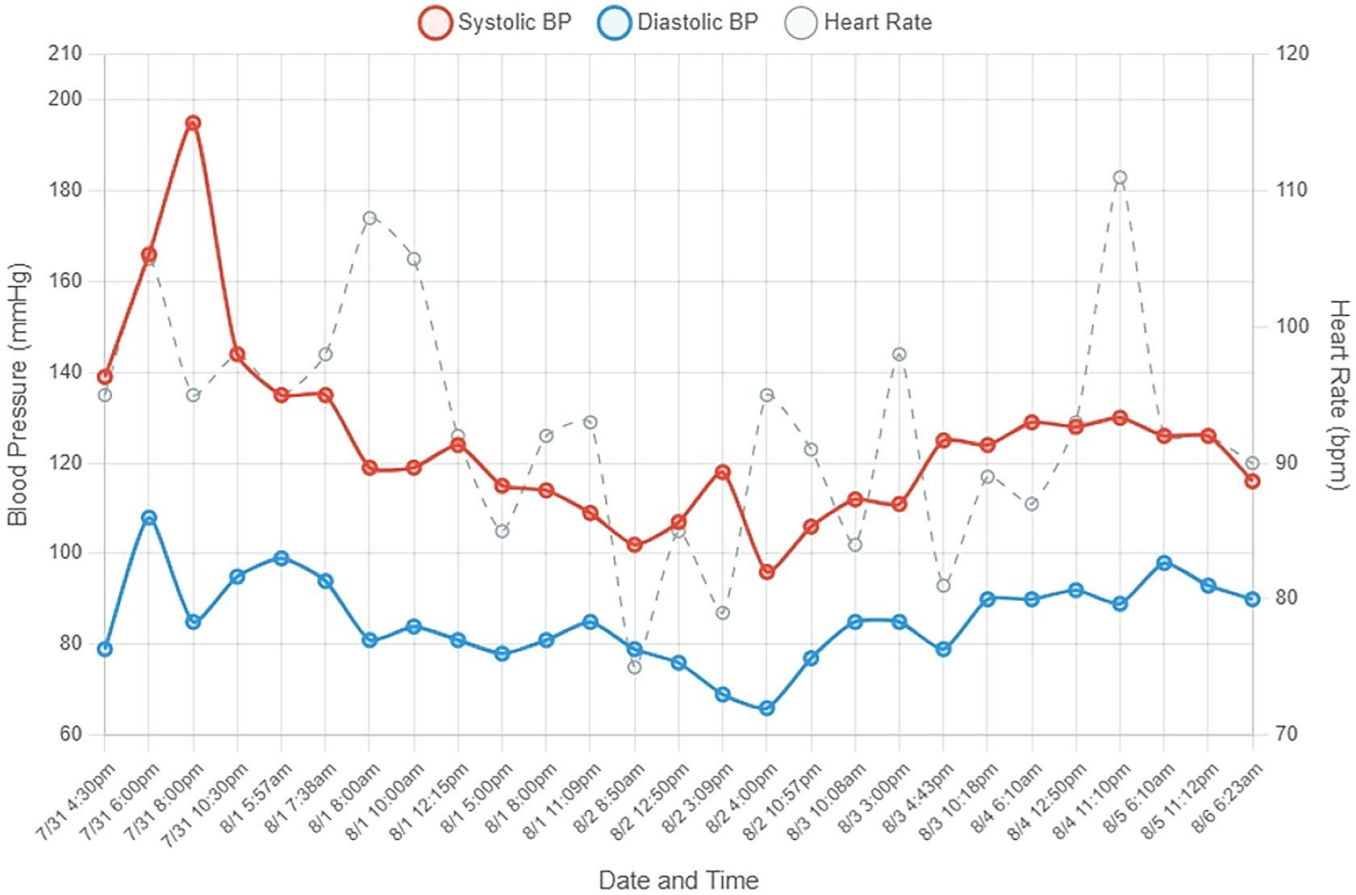
Home Blood Pressure readings after initiating the regimen. Home blood pressure and heart rate measurements recorded during FOLFIRINOX therapy demonstrate episodic hypertension temporally associated with chemotherapy administration. Hypertensive episodes occurred on chemotherapy days (7/31, 8/1), with peak values reaching 195/85 mmHg on 7/31, requiring medical intervention. Blood pressure returned to normotensive ranges during inter‐cycle periods (8/2 through 8/6). BP, blood pressure; HR, heart rate. X axis represents chronological dates and times. Y axis represents blood pressure (systolic and diastolic) in mmHg and heart rate in beats per minute.

Reviewing her detailed home blood pressure logs cemented the direct, temporal link between the hypertensive spikes and her chemotherapy administrations (Figures 1 and 2). With other causes excluded and this clear pattern established, we diagnosed her with chemotherapy‐induced hypertension.

## 4 Treatment

Following the hypertensive episode after cycle 2, the patient was initiated on losartan 50 mg daily. This regimen attenuated the infusion‐day hypertensive spikes; however, subsequent review of her ambulatory blood pressure logs revealed the emergence of symptomatic hypotension during the inter‐cycle period, with systolic readings frequently falling below 100 mmHg. This created a therapeutic dilemma because the dose required to control infusion‐related hypertension produced symptomatic hypotension between treatment cycles. The losartan dose was therefore reduced to 25 mg daily, which provided satisfactory baseline control without inter‐cycle symptoms. However, during cycle 10, despite being on losartan 25 mg daily, she had an hypertensive episode.

After this episode despite baseline losartan 25 mg therapy, a chronotherapeutic antihypertensive strategy was introduced. The patient received a single 5 mg dose of amlodipine 8–12 h prior to each scheduled FOLFIRINOX infusion. This timing was selected to coincide with the drug's peak pharmacodynamic effect. The patient was extensively counseled on holding all antihypertensive agents for systolic BP < 100 mmHg.

Because this chronotherapeutic antihypertensive strategy represents an off‐label and non–guideline‐directed approach for managing chemotherapy‐associated hypertension, a detailed informed consent discussion was conducted with the patient prior to implementation. The discussion addressed the rationale for timed antihypertensive administration, the risks of hypotension or inadequate blood pressure control, alternative strategies including escalation of baseline antihypertensive therapy or modification of the chemotherapy regimen, and the anticipated benefit of preventing infusion‐related hypertensive crises while preserving treatment continuity. The patient expressed significant apprehension regarding the recurrent blood pressure spikes and strongly preferred a strategy that would proactively prevent infusion‐related hypertensive episodes. A structured monitoring plan including home blood pressure logging and clear thresholds for medication withholding was also established prior to initiation.

## 5 Outcome and Follow up

This two‐pronged strategy proved highly effective. The patient achieved hemodynamic stability throughout her treatment cycles. During subsequent infusions with the preemptive amlodipine regimen, systolic blood pressure remained approximately 125–145 mmHg and diastolic pressure 75–90 mmHg during chemotherapy sessions. The pre‐emptive amlodipine successfully blunted the hypertensive surges during infusions, with no further crises or requirement for acute intervention. Critically, this tailored approach permitted the uninterrupted administration of full‐dose FOLFIRINOX, which was associated with a continued profound radiographic and serologic treatment response.

**FIGURE 3:**
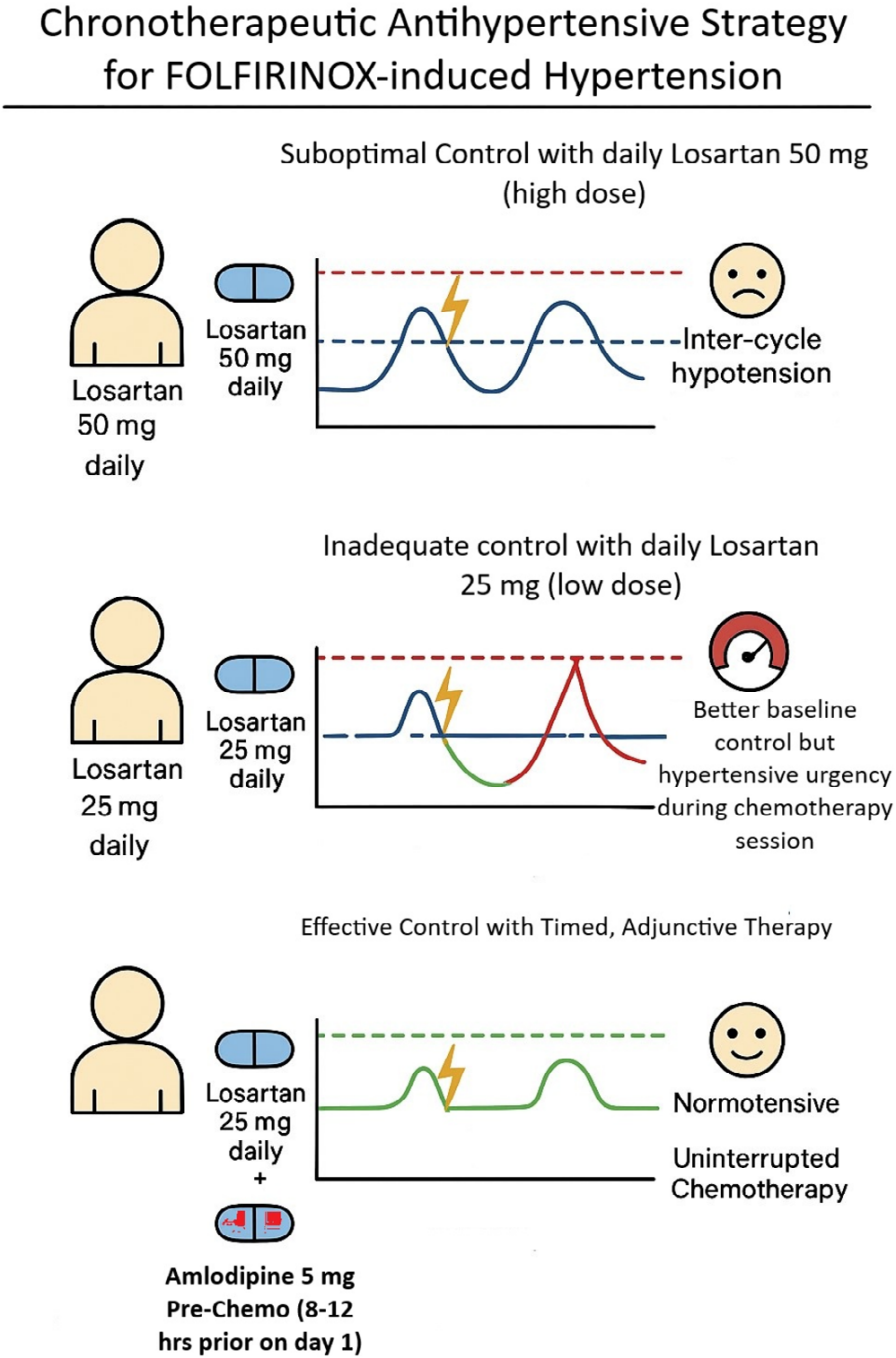
Graphical illustration of chronotherapeutic antihypertensive strategy for FOLFIRINOX‐induced hypertension. Pre‐emptive amlodipine 5 mg administered 8–12 h before infusion combined with daily losartan 25 mg achieved hemodynamic stability while avoiding inter‐cycle hypotension.

After completing 11 cycles of full‐dose FOLFIRINOX with sustained clinical benefit, her regimen was modified to mitigate cumulative neurotoxicity; oxaliplatin was discontinued, and she continued on maintenance therapy with irinotecan, 5‐fluorouracil, and leucovorin (modified FOLFIRI). Recent imaging shows her disease remains stable, her CA 19–9 continues to trend favorably, and she maintains her functional independence. She has developed mild tingling in her right foot (possibly early neurotoxicity), for which we are checking vitamin B12, folate, magnesium, and phosphorus levels. Otherwise, she reports no significant new gastrointestinal or constitutional symptoms. Our chronotherapeutic antihypertensive strategy is summarized in Figure 3.

## 6 Discussion

This case highlights an underrecognized challenge in the management of CTIH during FOLFIRINOX treatment. Analysis of available clinical trial data reveals substantial heterogeneity in both the recognition and reporting of CTIH. Incidence estimates ranging from 11% to 22% across trials (NCT03099265, NCT01688336, NCT01959139) likely reflect fundamental differences in blood pressure monitoring protocols, varying definitions of clinically significant hypertension (Grade 2 vs. Grade 3 events), and heterogeneous baseline cardiovascular risk profiles among enrolled patients. The absence of standardized monitoring and reporting criteria (NCT01926197) limits the ability to accurately quantify the true scope of this problem and to develop evidence‐based management strategies.

The immediate clinical consequences of uncontrolled CTIH extend beyond the risk of hypertensive emergency and target‐organ damage. An additional and clinically important consequence involves disruption of optimal chemotherapy delivery; infusions may be interrupted or halted for urgent blood pressure management, resulting in suboptimal drug exposure, altered pharmacokinetics, and potentially compromised treatment efficacy. In a disease where therapeutic windows are narrow and treatment options limited, any reduction in chemotherapy delivery intensity may adversely affect survival outcomes.

Our patient's episodic hypertensive pattern, severe blood pressure elevations temporally associated with FOLFIRINOX infusions with inter‐cycle normotension, suggested a direct treatment‐related effect rather than progression or exacerbation of underlying hypertension. Comprehensive evaluation excluded secondary causes including pheochromocytoma, renovascular disease, and medication‐induced hypertension from other agents. The failure of maximally tolerated losartan therapy to prevent these episodes, combined with problematic inter‐cycle hypotension, necessitated an alternative therapeutic strategy.

The pharmacokinetic profile of amlodipine is well suited to this clinical scenario. With peak plasma concentrations achieved approximately 6–12 h after administration and an elimination half‐life of 30–50 h, a single pre‐infusion dose can achieve maximal vasodilatory effect during peak chemotherapy exposure. The drug's long half‐life helps maintain stable plasma levels that reduce the risk of rebound hypertension, while its gradual offset limits abrupt hemodynamic changes during the inter‐cycle period. When administered 8–12 h prior to FOLFIRINOX infusion, amlodipine effectively attenuated hypertensive surges without causing clinically significant inter‐cycle hypotension.

Current literature often emphasizes angiotensin receptor blockers (ARBs), particularly losartan, as preferred agents for managing CTIH in this population [9, 10]. However, comparative effectiveness data among antihypertensive classes remain limited, creating clinical equipoise when patients do not tolerate or respond to ARB therapy. Our successful use of timed calcium‐channel blockade suggests that aligning pharmacologic intervention with the temporal pattern of hypertensive episodes may be more important than drug class selection alone.

The mechanistic basis for this approach may extend beyond simple vasodilation. Amlodipine's effects on endothelial function, including enhanced nitric oxide bioavailability and reduced oxidative stress, may counteract chemotherapy‐associated vascular dysfunction. Additionally, its anti‐inflammatory properties, mediated through suppression of NF‐κB signaling and reduced expression of adhesion molecules, could theoretically mitigate inflammatory pathways triggered by components of the FOLFIRINOX regimen [11, 12].

To our knowledge, this represents the first reported description of prophylactic pre‐infusion amlodipine used specifically to prevent FOLFIRINOX‐associated hypertensive crises [13, 14]. While previous studies have investigated calcium‐channel blockers as potential chemotherapy‐sensitizing agents, none have explored their role in targeted CTIH prevention through chronotherapeutic dosing strategies. The success of this strategy, evidenced by sustained hemodynamic stability across multiple treatment cycles and the ability to continue both standard and maintenance chemotherapy without interruption, suggests a potentially valuable therapeutic approach. However, its applicability remains limited, as these findings derive from a single case and cannot be broadly generalized without further investigation.

Several limitations merit consideration. As a single case report, generalizability is inherently limited. We lack pharmacokinetic monitoring to confirm optimal drug levels coinciding with peak hypertensive risk. Ambulatory blood pressure monitoring could provide more granular data regarding circadian patterns and treatment response. The absence of biomarkers for endothelial dysfunction or inflammatory activation limits mechanistic insight. Furthermore, it is not possible to distinguish whether improved outcomes were attributable to improved chemotherapy delivery or to any potential direct antitumor effects of antihypertensive therapy without controlled studies.

Emerging evidence suggests antihypertensive agents, including losartan and amlodipine, have been hypothesized to influence oncologic outcomes in pancreatic adenocarcinoma through mechanisms independent of blood pressure control [9, 10, 13, 14, 15]. Proposed mechanisms include VEGF pathway suppression, modulation of tumor–stromal interactions, and enhancement of drug delivery through improved tumor perfusion. However, the evidence supporting these effects is largely derived from retrospective analyses and preclinical studies, and no causal relationship has been established in clinical practice.

Future investigations should address several key questions. A phase II study comparing prophylactic versus reactive antihypertensive strategies, stratified by baseline cardiovascular risk and FOLFIRINOX dose intensity, could help define optimal management approaches. Pharmacokinetic studies correlating antihypertensive drug levels with blood pressure response may further refine dosing strategies. Biomarker development to identify patients at highest risk for CTIH could enable targeted prevention. Investigation of whether this chronotherapeutic approach applies to other chemotherapy regimens associated with acute or infusion‐related hypertension also warrants exploration.

The broader implications extend beyond FOLFIRINOX. If validated in prospective studies, timed antihypertensive administration may represent a practical strategy for managing CTIH. This approach acknowledges that chemotherapy‐associated cardiovascular toxicity may follow predictable temporal patterns that are amenable to targeted intervention, rather than requiring continuous antihypertensive therapy that may worsen inter‐treatment hypotension.

In conclusion, this case highlights a previously underrecognized complication of FOLFIRINOX therapy: acute, infusion‐associated hypertensive crises, and demonstrates the feasibility of a chronotherapeutic antihypertensive strategy tailored to the temporal pattern of blood pressure elevation. Timed pre‐infusion administration of amlodipine successfully prevented hypertensive surges while preserving inter‐cycle hemodynamic stability, enabling uninterrupted chemotherapy delivery. However, as a single‐patient observation, these findings should be considered hypothesis‐generating and not indicative of established clinical efficacy or generalizable practice. This approach underscores the potential value of integrating chronotherapeutic principles into oncology supportive care, particularly in the management of treatment‐related cardiovascular toxicities. Prospective studies are required to validate this strategy, define optimal timing and dosing parameters, and determine its applicability across broader patient populations and chemotherapy regimens.

## Author Contributions

**Manas Pustake:** conceptualization, data curation, formal analysis, funding acquisition, investigation, methodology, project administration, resources, software, supervision, validation, writing – original draft, writing – review and editing. **William Wells‐Gatnik:** conceptualization, data curation, formal analysis, funding acquisition, investigation, methodology, project administration, resources, software, supervision, validation, visualization, writing – original draft, writing – review and editing. **Vani Shukla:** conceptualization, data curation, formal analysis, investigation, methodology, resources, software, supervision, validation, writing – original draft, writing – review and editing. **Javier Corral:** conceptualization, data curation, formal analysis, investigation, methodology, resources, supervision, validation, visualization, writing – original draft, writing – review and editing. **Sumit Gaur:** conceptualization, data curation, formal analysis, investigation, methodology, resources, software, supervision, validation, visualization, writing – original draft, writing – review and editing.

## Funding

The authors have nothing to report.

## Consent

Written informed consent was obtained from the patient for publication of this case report and any accompanying figures or clinical data. All identifying information has been anonymized to protect patient privacy in accordance with the Declaration of Helsinki.

## Conflicts of Interest

The authors declare no conflicts of interest.

## Data Availability

Data about patient's clinical profile will be available upon request, maintaining the anonymity.
